# Adult Male Mice Emit Context-Specific Ultrasonic Vocalizations That Are Modulated by Prior Isolation or Group Rearing Environment

**DOI:** 10.1371/journal.pone.0029401

**Published:** 2012-01-06

**Authors:** Jonathan Chabout, Pierre Serreau, Elodie Ey, Ludovic Bellier, Thierry Aubin, Thomas Bourgeron, Sylvie Granon

**Affiliations:** 1 Centre de Neuroscience Paris Sud, Team “Neurobiologie de la Prise de Décision”, Université Paris Sud 11 & CNRS UMR 8195, Orsay, France; 2 Institut Pasteur, “Neurobiologie Intégrative des Systèmes Cholinergiques” Unit, CNRS URA 2182, Paris, France; 3 Institut Pasteur, “Génétique Humaine et Fonctions Cognitives” Unit, CNRS URA 2182, Paris, France; 4 Centre de Neuroscience Paris Sud, Team “Communication Acoustique”, Université Paris Sud 11 & CNRS UMR 8195, Orsay, France; Case Western Reserve University, United States of America

## Abstract

Social interactions in mice are frequently analysed in genetically modified strains in order to get insight of disorders affecting social interactions such as autism spectrum disorders. Different types of social interactions have been described, mostly between females and pups, and between adult males and females. However, we recently showed that social interactions between adult males could also encompass cognitive and motivational features. During social interactions, rodents emit ultrasonic vocalizations (USVs), but it remains unknown if call types are differently used depending of the context and if they are correlated with motivational state. Here, we recorded the calls of adult C57BL/6J male mice in various behavioral conditions, such as social interaction, novelty exploration and restraint stress. We introduced a modulator for the motivational state by comparing males maintained in isolation and males maintained in groups before the experiments. Male mice uttered USVs in all social and non-social situations, and even in a stressful restraint context. They nevertheless emitted the most important number of calls with the largest diversity of call types in social interactions, particularly when showing a high motivation for social contact. For mice maintained in social isolation, the number of calls recorded was positively correlated with the duration of social contacts, and most calls were uttered during contacts between the two mice. This correlation was not observed in mice maintained in groups. These results open the way for a deeper understanding and characterization of acoustic signals associated with social interactions. They can also help evaluating the role of motivational states in the emission of acoustic signals.

## Introduction

Social approach is one of the most basic behavioral components of all social interactions. However, the initial motivation to approach a conspecific may be independent of territory defence or mating behavior. This has been shown in juvenile mice [1] and in adult male-male interactions, when occurring in a novel environment [2], [3].

One of the critical questions is to determine what the motivations behind such social approaches are and which behavioral and neurobiological mechanisms support them. Indeed, it remains currently unclear whether social approach or social proximity, when not associated to territory defence and a reproductive motivation, also impart a reward value [4].

Social interactions are frequently associated with species-specific vocalizations to transmit different types of information that may concern individual characteristics (age, sex, body size), but also the individual's emotional states and/or its social status. Rodents emit ultrasonic vocalizations -USVs- that have been mostly studied in pups as a response to maternal separation [5], or stressor exposure [6], [7], and in adults in response to stressful [8], [9], or pleasurable events [8], [10]. Furthermore, in rats, high frequency modulated USVs carry reward-related and positive social information [10]. This issue is nevertheless much less studied in mice and thus debatable [11]. Although it has long been believed that USVs are not emitted during male-male agonistic encounters, a recent study showed that, when placed in the home cage of another male, C57BL/6 male mice emit different types of USVs [12]. During the brief period of the resident-intruder experiment (3 minutes), a social hierarchy between the two animals was not established. Dyads were involved in “affiliative behaviors” with concomitant emission of USVs, although this task is highly stressful for intruders [13].

When animals are not focused on behavioral responses leading to reinforcements, which would be the case in conditioning or in spatial learning tasks, it is difficult to capture and identify which element(s) in the environment they use to make choices. In order to identify such elements we previously designed a social interaction task in which we can manipulate two competing motivations: the one for social contact and the one for novelty exploration [2], [3], [14], [15]. This experimental protocol allows the study of the establishment of social interactions in adult males, without any notion of mating and with limited aggressiveness, if any. The aim of the present study is to address how behavioral context influences the vocal behavior of adult male mice during social interaction tasks (SIT) and non-social tasks (novelty exploration and restraint stress). We also evaluated the impact of social reward modulation by prior isolation or group housing on USVs features.

## Materials and Methods

### Ethics Statement

The animals were treated according to the ethical standards defined by the Centre National de la Recherche Scientifique for animal health and care in strict compliance with the EEC recommendations (n°86/609). All efforts were made to minimize animal discomfort and to reduce the number of animals used. We tested 64 C57BL/6J mice purchased from Charles Rivers Laboratories France (L'Arbresle Cedex, France). They were 11 to12 weeks old at their arrival and remained stabulated in a standard rearing facility in collective cages (4 to 5 animals per cage) during one week before any experiment. Room ventilation, temperature and humidity were controlled with a 12/12 light-dark cycle (light on at 8:00 am). They received standard chow and water *ad libitum*. For the two “isolated” experimental conditions mice were thereafter placed in individual cages three weeks before the experiment while animals from other experimental groups remained in collective cages.

### Behavioral procedures

#### Behavioral apparatus & protocols

We combined the recording during SIT and novelty exploration within the same protocol as described hereafter. The SIT condition was conducted as described previously [2], [14]. Animals were 15 weeks old at testing day. The day of the experiment each animal was allowed to visit alone the novel environment for 30 minutes consisting of a transparent Plexiglas cage containing fresh bedding (50 cm×30 cm×30 cm) placed in an unfamiliar quiet room. The experimental cage was situated on a table, under a numeric video camera connected (Hercules®) to a computer (recording at 33 frames per sec). Light was set at 100 Lux by undirected bulbs. USVs were recorded during the first 4 minutes of this exploration/habituation phase (“exploration” condition). After habituation, the “visitor” animal was gently introduced in the cage. “Visitors” were male mice unknown from the tested mouse, of the same age from the same strain. “Visitors” had always been maintained in social cages. Frequency and duration of social contacts and USVs were recorded for 4 minutes. At the end of the experiment, animals were placed back in their respective home cage. Each dyad was used only once. The restraint stress condition (“restraint” condition) was also conducted in an unfamiliar quiet room. Mice were placed for 10 minutes in a 50 ml Falcon® tube opened at one end to allow breathing. The Falcon® tube was fixed in an empty opaque cage to avoid rolling movement. In all conditions, the experimenter was out of sight of the animals.

**Figure 1:**
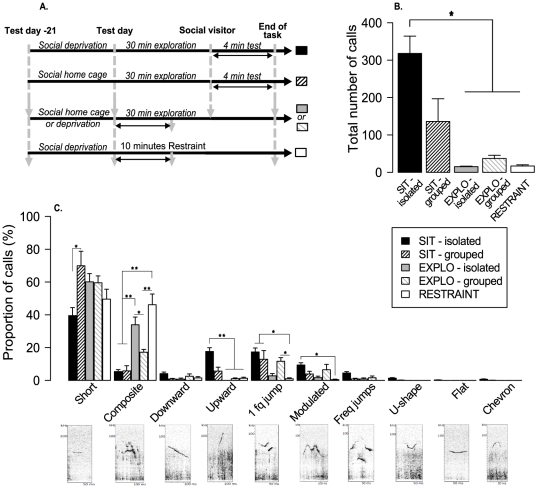
Number of calls and vocal repertoire uttered in five behavioral contexts. A- Calls were recorded during social interaction task, novelty exploration, and restraint stress and analyzed off line. B- Total number of calls emitted in 4 minutes: SIT-isolated (n = 17), SIT-grouped (n = 8), EXPLO-isolated (n = 15), EXPLO-grouped (n = 8) and RESTRAINT (n = 16). C- Distribution and **s**pectrograms of the ten call types typically emitted by adult male mice. Data not shown, proportion of “other” calls: 1.4±0.8% in SIT-isolated, 4.5±1.3% in SIT-grouped, 9.5±4.1% in EXPLO-grouped, 3.1±1.3% in EXPLO-isolated and 16.3±3.1% in Restraint stress. (Time and frequency criterion were used to distinguish these categories, *see methods*). Data are presented as means ± SE. *: p<0.005; **: p<0.0001 for chi-square and Mann-Whitney paired comparisons.

Five conditions were then examined in five independent groups (Figure 1A):

1- SIT with mice isolated before the experiment (“SIT-isolated”, n = 17)

2- SIT with mice maintained in group (4 animals per cage) before the experiment (“SIT-grouped”, n = 8)

3- Novelty exploration with: mice isolated before the experiment (“Explo-isolated”, n = 15)

4- Novelty exploration with mice maintained in group (4 animals per cage) before the experiment, (“Explo-grouped”, n = 8).

5- Restraint stress with mice maintained in group (4 to 5 animals) cages (“Restraint”, n = 16).

#### Behavioral analysis in SIT

In SIT condition, we scored the duration and number of social contacts and analysed the behavioral sequences between the two conspecifics for four minutes. Within the social contacts, we discriminated different subtypes: Oro-oral, oro-flank, oro-genital and others (which included flank-flank, genital-genital).

#### Ultrasonic vocalization Recording

A condenser ultrasound microphone Polaroid/CMPA was placed above the experimental chamber, high enough so that the receiving angle of the microphone covered the whole area of the test cage or attached to a tripod in front of the tube containing the restrained mice. It was connected to an ultrasound recording interface Ultrasound Gate 416H, which was itself plugged into a personal computer equipped with the recording software Avisoft Recorder USG (Sampling frequency: 250 kHz; FFT-length: 1024 points; 16-bits). All recording hardware and software were from Avisoft Bioacoustics ® (Berlin, Germany).

#### Acoustic variables

For all behavioral conditions USVs were analysed off line with SASLab Pro (Avisoft Bioacoustic ®, Berlin, Germany). Spectrograms were generated for each detected call (Sampling frequency: 250 kHz; FFT-length: 1024 points; 16-bit; Blackman window; overlap: 87.5%; time resolution: 0.512 ms; frequency resolution: 244 Hz). Audio recordings were disturbed by the background noise originating from the animals moving and/or digging in the fresh bedding. We nevertheless kept the bedding because social interactions may have been affected by its absence and we wanted to match as closely as possible to our classical experimental conditions [2].

We recorded the total number of calls emitted by each pair of mice during SIT, and manually measured different variables related to peak frequency (Pf_start_ [peak frequency at the beginning of the call], Pf_end_ [peak frequency at the end of the call], Pf_min_ [minimum peak frequency], Pf_max_ [maximum peak frequency]) for each call. We categorized the waveform pattern of each call as belonging to one of ten distinct categories based on their duration and frequency modulation (adapted from [5], [8], [12], [16]). We calculated the proportion of each call category for each pair of mice in SIT and for each individual mouse in other conditions.

The ten categories illustrated in Figure 1C were:

1. Short: ≤50 ms and ≤10 kHz frequency modulation.
2. Flat: ≥50 ms and ≤10 kHz frequency modulation.
3. One frequency jump: instantaneous frequency step, like a vertical discontinuity with no time gap.
4. Multiple frequency jumps: multiple instantaneous frequency step.
5. U: U-shape wave ≥10 kHz frequency modulation.
6. Chevron: inverted-U shape ≥10 kHz frequency modulation.
7. Modulated: ≥10 kHz of modulation, several decreases and increases in frequency.
8. Composite: two or more components emitted simultaneously.
9. Upward: continuous increase in peak frequency ≥10 kHz frequency modulation.
10. Downward: continuous decrease in peak frequency ≥10 kHz frequency modulation.

#### Synchronization of audio and video files

We performed a “clap” with finger in the field of the camera to time-matched video and audio files. In the audio files, we cut the information before this sound, and in the video files we selected the exact frame of this event and started from this point. This manual synchronization permitted us to analysed which USVs were emitted during contact and non-contact events. We then further classified contact events in four categories (see above): Oro-oral, Oro-flank, Oro-genital and others.

### Statistical analyses

Kruskall & Wallis non parametric tests were used for behavioral variables and USVs quantitative (number of calls) and qualitative (acoustic variables) variables in each of the five conditions. Correlation data were analysed with a Spearman correlation test between behavioral measures and number of calls uttered within each category. Dependent variables (contact *versus* non-contact repartitions of calls) were analysed with Wilcoxon signed-rank test. The significance threshold was set at p<0.05. Post Hoc comparisons were performed using Mann-Whitney non parametric tests or Chi-square test (noted X^2^) only when appropriate (Statview software; computing and statistical software R [17]. For all post-hoc paired comparisons a Bonferroni correction was applied because of the number of test repetitions, therefore setting the significance threshold at p<0.005.

## Results

In the current experiments, all mice emitted USVs in all conditions.

### Mouse USV emissions are context dependent

We first quantified the amount of USVs emitted in the five different conditions tested. There was a major group effect in the number of calls emitted by adult male mice (**Figure 1B** **& Table S1A;** H 4 = 34.303, P = <0. 0001). Post-hoc comparisons showed that mice in both SIT conditions emitted significantly more calls than mice in all other conditions (Table S1). In social context, mice previously isolated (SIT-isolated) tended to emit more calls than mice housed in groups (SIT-grouped), but it was not significant after correction for multiple testing (U = 29.5, P = 0.024).

We then examined the composition of the vocal repertoire in the five behavioral conditions. In all conditions, a limited proportion of call-like spectrograms (“others”) could not be classified because of the background noise and a non discriminable shape. These “calls” were thus not included in the calculation and analyses performed thereafter (**Figure 1C** **& Table S1 B–K**).

Male mice emitted a large variety of calls during social and exploratory behaviors, while restraint mice show a narrower repertoire, principally composed of “short” and “composite”. Indeed restraint mice emitted more “composite” than both SIT mice (For SIT-isolated: X^2^ = 38.53, P<0.0001, for SIT-grouped: X^2^ = 31.87, P<0.0001) and EXPLO-grouped (X^2^ = 17.22, P<0.0001), less “upward” than SIT-isolated (X^2^ = 14.14, P<0.0001), less “one frequency jump” than both SIT (SIT-isolated: X^2^ = 14.99, P = 0.0001, SIT-grouped: X^2^ = 10.07, P = 0.0015), and less “modulated” than SIT-isolated (X^2^ = 8.11, P = 0.004).

“Short” calls were the most uttered calls for all experimental conditions. SIT-isolated mice emitted less “short” than SIT-grouped (**Figure 1C**: X^2^ = 8.46, P = 0.003) and used a largest vocal repertoire (i.e. all call types were represented). Specifically, SIT-isolated had a higher proportion of “upward” (compared with EXPLO-isolated: X^2^ = 17.62, P<0.0001; EXPLO-grouped: X^2^ = 14.87, P = 0.0001 and Restraint: X^2^ = 14.14, P = 0.0001), “one frequency jump” (for Restraint; X^2^ = 14.99, P = 0.0001), “modulated” (for Restraint; X^2^ = 8.11, P = 0.004). Additional categories of calls like “upward”, “flat”, “chevron” and “U-shape” emerged and the proportion of “composite” calls was relatively low (**Figure 1C** **& Table S1 B–J**). In some contexts, several call types were completely absent. For instance, “flat”, “chevron” and “U-shape” call types were not recorded during exploration (whether animals were isolated or not), and “multiple frequency jumps”, “U-shape”, “flat” and “chevron” calls were not recorded in the restraint condition. Interestingly, the repertoire did not vary significantly according to the housing condition (isolated vs. grouped), except for “short” during SIT and “composite” during Exploration.

### Mouse USV acoustic features are context dependent

**Figure 2:**
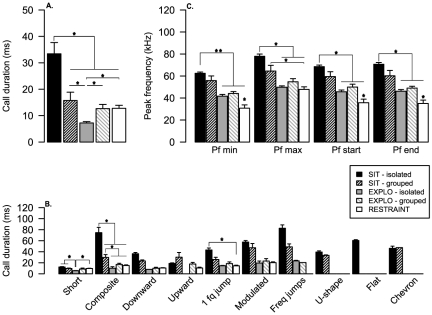
Acoustic characteristics of calls emitted in five behavioral contexts. A- Calls durations in all conditions. B- Calls durations for all call types in all conditions. C- Frequency features (“min”, “max”, “start” and “end” frequencies) of calls in all conditions. Data are presented as means ± SE. *: p<0.005; **: p<0.0001 for Mann-Whitney paired comparison.

In order to better characterize the acoustic features of the USVs we analysed in depth their duration and their peak frequency related variables. There was a significant condition effect in the total call duration (group effect, H4 = 36.51; p<0.0001; **Figure 2A** **& Table S2 A**). Call duration was the longest for SIT-isolated mice (U = 112; p<0.0001 for SIT-grouped, U = 120; p = 0.00147 for Explo-isolated, U = 245; p<0.001 for Explo-grouped and U = 234; p = 0.002 for restraint) and the shortest for Explo-isolated mice (U = 112; p = 0.0002 for SIT-grouped, U = 16; p<0.001 for Explo-grouped and U = 18; p<0.0001 for Restraint; **Table S2A**). Since this result might be due to presence of long calls emitted by SIT-isolated mice, we investigated in more details the duration of each call type separately to take into account the effect of the vocal repertoire. SIT-Isolated mice showed longer “short” calls than EXPLO-isolated (U = 248; p<0.0001), longer “composite” calls than all the other condition (U = 93.5; p = 0.0042; for SIT-grouped, U = 118; p<0.0001 for EXPLO-grouped, U = 221; p<0.0001 for EXPLO-isolated, U = 238; p<0.0001 for Restraint), and longer “one frequency jump” than EXPLO-grouped (U = 101; p = 0.0014) (**Figure 2B** **& Table S2 B–F**).

There was a group effect in the peak frequency (Pf min: H 4 = 42.82, P = <0.0001; Pf max: H 4 = 38.47, P = <0.0001; Pf start: H 4 = 42.34, P = <0.0001; Pf end: H 4 = 44.50, P = <0.0001, **Figure 2C** **& Table S3 A–D**), which was still valid when calls types were considered separately (“short” and “composite” calls; **Figure S1**). Mice tested in SIT emitted calls between 62 kHz+/−1.3 (Pf min) and 77 kHz+/−1.8 (Pf max) with a significant difference between “SIT-isolated” and exploration or restraint condition for all parameters (**Table S3 A& B**), whereas mice exploring the novel environment emitted calls between 40 kHz+/−2.4 (Pf min) and 55 kHz+/−3.5 (Pf max) with no significative difference between “Explo-isolated” and “Explo-grouped” conditions. Mice in restraint stress condition emitted calls between 30 kHz+/−2.7 (Pf min) and 48 kHz+/−1.8 (Pf max). For minimum peak frequency mice in restraint stress condition emitted calls significantly lower than all other conditions (see **Table S3 A–D**). Housing condition did not influence the peak frequency variables for mice tested during social interaction or exploration (see **Table S3**).

### Isolation or group housing influences social and vocal behaviors

**Figure 3:**
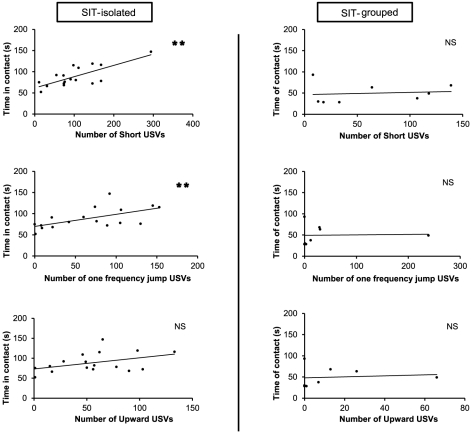
Correlation between behavioral contexts and calls emission. Correlation between the number of calls of the 3 main categories (“Short”, “Jump” and “Upward”) and duration of contact during social interaction in isolated or non-isolated mice. **: p = 0.005 for Spearman rank correlation test and NS: p>0.05.

We analysed the correlation between the emission of the calls and the social interactions in SIT-isolated and SIT-grouped mice. The total number of calls and duration of social contacts were significantly and positively correlated in the SIT-isolated condition (r s = 0.778, n = 17, P = 0.0001) but not in the SIT-grouped condition (r s = 0.102, n = 8, P = 0.81). In the remaining analyses we focused on the most uttered calls, namely “short”, “one frequency jump” and “upward” calls. For mice in SIT-isolated condition, there was a strong positive correlation between the duration of social contacts and the number of “short” and “one frequency jump” calls (respectively r s = 0.749, n = 17, P = 0.0003; r s = 0.601, n = 17, P = 0.0094). In contrast, in SIT-grouped condition there was no evidence for correlation between these call types and the duration of social contact (“Short”: r s = 0.120, n = 8, P = 0.787, NS; “one frequency jump”: r s = 0.044, n = 8, P = 0.922, NS; “Upward”: r s = 0.119, n = 8, P = 0.788, NS) (**Figure 3**).

**Figure 4:**
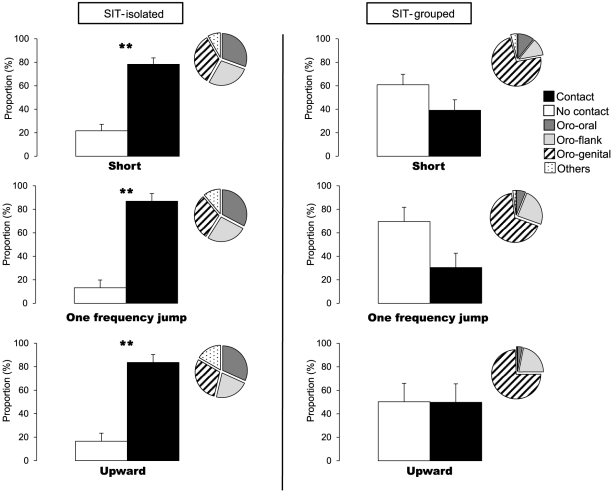
Behavioral contexts associated with calls emission during social interactions in isolated and non-isolated mice. Proportion of calls emitted during contacts and independently of social contact. Inset proportion of social contacts types (oro-oral sniffing, oro-flank sniffing, oro-genital sniffing and other contact) associated with the emission of the 3 main calls types (short, jump and upward). Data are presented as Means ± SE, **: p<0.005 for Wilcoxon signed-rank test.

We then examined more closely the behaviors in which “short”, “one frequency jump” and “upward” calls were uttered. SIT-isolated mice emitted the majority of their calls during contact (**Figure 4**, left panel): Approximately 80% of “short”, “one frequency jump” and “upward” call types were uttered during contact while only 20% were emitted when both mice were apart (respectively z = −3.24, P = 0.0012; z = −2.82, P = 0.0048; z = −2.84, P = 0.0045, **Figure 4**, left panel). A deeper analysis of the types of contact showed that mice previously isolated uttered the 3 main call types equally during oro-oral, oro-side and oro-genital contacts.

We did not observe the same distribution in SIT-grouped mice (**Figure 4**, right panel) since “short”, “one frequency jump” and “upward” calls were emitted mostly when mice were apart (“short”: z = −1.09, P = 0.271; “one frequency jump”: z = −1.36, P = 0.173; “upward”: z = −0.73, P = 0.463, **Figure 4**, right panel). During contact more than 50% of the calls were uttered during oro-genital sniffing.

## Discussion

The present study investigates in details the vocal behavior of adult male mice during a same-sex social interaction task, as well as in two non-social tasks (i.e., novelty exploration and restraint stress). We first showed that the number of calls emitted as well as a number of frequency parameters varied according to the behavioral context of emission. As expected, adult male mice emitted USVs in the social interaction task, but, remarkably, they also emitted some USVs in non-social tasks. Finally, housing conditions, which act as a modulator for social motivation, also appeared to influence vocal behavior during social interactions task and more unexpectedly also during exploration task.

### Like rats, mice emit context-specific USVs

It's commonly known that rodents emit vocalizations particularly in presence of conspecifics [1], [5], [10], [12], [18]–[22]. These paradigms are, for the most of them, associated to a social condition with direct cues (presence of conspecifics) or indirect cues (urine presentation, playback emission). It was suggested in the literature that mice would not produce USVs during aversive situations such as physical restriction or electric shock [11]. However in rats, several authors reported the recording of calls during various non-social conditions, including aversive situations or aggressive behavior [23]–[27]. Here we report for the first time in adult male mice the recording of USVs in different non-social contexts, such as exploration of a novel environment or restraint stress. Indeed male mice emitted calls during novelty exploration, whether they were previously maintained isolated or not, even though the total number of calls emitted during exploration was significantly lower than during SIT. The function of these USVs in novelty exploration remains to be investigated (related with a rewarding behavior, expression of anxiety…). It might be interesting to examine these calls in mouse models for autism spectrum disorders or for speech disorders (such as *FoxP2* mice for example, see [16]). Comparing the emission of such apparently “non-social” USVs between mutant and wild-type mice should allow differentiating between global impairments in emission of all types of ultrasonic signals and specific deficits in the emission of social ultrasonic signals.

**Figure 5:**
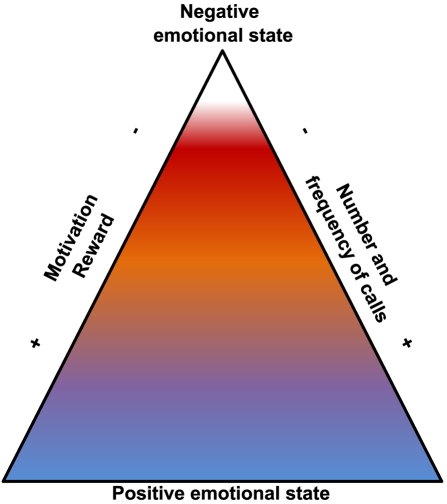
Interpretative schema of involvement of calls in motivational/emotionnal process. Proposition of the link between parameters of the calls and motivational/emotionnal processes in adult male mice.

The amount of calls was different between non-social and social situations. The social interaction task triggered the largest amount of USVs, which represented much more calls than the double of those recorded in the exploration or restraint conditions. This suggests that we did not simply record more calls because we tested two mice together. In SIT, we recorded from a dyad of mice, and thus cannot take for certain that only the isolated mouse vocalized. However, the restraint stress condition elicited very few calls, while the novelty exploration condition elicited an intermediate number of calls. This increasing number of calls emitted might reflect the increasing positive emotional state in these contexts, from a stressful situation (restraint stress; negative emotional state), to a neutral context eliciting limited anxiety (novelty exploration), and to a context eliciting a positive emotional state (social interactions; Figure 5). These results suggest that mice's USVs are used predominantly as social signals, but they can be secondarily used in contexts not directly involving a conspecific. Whether the later USVs are still directed to a potentially remote conspecific (despite the limited propagation of USVs at a relatively high frequency) remains to be examined.

We also showed that the vocal repertoire was different between paradigms. Mice uttered “short” and “composite” calls in all paradigms. However, mice in the SIT used a richer repertoire, with more “upward”, “multiple frequency jumps”, “U-shape”, “flat” and “chevron” calls than mice in the other non-social paradigms. “short” and “composite” calls may therefore represent “basic” call types found in any behavioral contexts, while other calls may be more “informative” of the behavioral, emotional or motivational content of a situation. In restraint stress, mice emit principally “basic” calls of lower frequency, which might be similar to the 22 kHz alarm calls emitted by rats in aversive context [23], [26], [28]. However, the 22 kHz calls of rats appeared more stereotyped (in terms of frequency modulations at least) [27] in comparison with the “basic” calls observed in restrained mice (without footshock), underlying subtle differences in these signals between rats and mice.

Calls emitted in a social interaction task were longer in comparison with calls emitted in other non-social situations. In addition, high frequency calls were recorded in the social interaction task, while the lowest frequency calls were recorded in the stressful situation. During signal evolution, different pressures may arise in signal design depending on whether it is specialized to advertise or privatize information [29]. Thus, it is possible that calls emitted in social situations correspond to a “private” information, transmitted at short-range, as high frequencies propagate less than lower ones. Conversely, calls emitted in stressful situations, lower in frequency, should be “public” signals advertising to a wide audience at long-range.

Therefore, these frequency changes dependent of the context seem to be common to different species in which the motivational or emotional states induce prosodic changes (e.g., rats, humans, birds [7], [10], [23], [24], [27], [28], [30]–[33]. In the present study, significant frequency variations were highlighted between positive and negative motivational states. Differences related to the gradation within a positive arousal state (high arousal/motivation after social isolation and lower arousal/motivation after group housing in the SIT) were more subtle but still significant, similarly to variations related to the degree of arousal in several species [34]–[36]. In these species, whether negative or positive, a higher arousal was related to higher frequency characteristics. Therefore, within a positive arousal state, the gradation is encoded similarly from mice to primates. The expression of arousal gradation in mice within a negative arousal state remains to be examined. These findings could then be applied in mouse models for autism spectrum disorders or speech disorders to check their abilities to encode arousal degree in their ultrasonic signals. In line with this reasoning, our results, obtained while recording a dyad of mice, suggest that it is mostly the mouse previously isolated -SIT isolated- which emit the most part of the USVs, as social contact constitutes a positive rewarding situation associated with high frequency calls. However, we cannot exclude the other mouse of the dyad to contribute to the USVs recorded.

### Past social experiences influence social interaction and vocalization behavior

We previously showed [3] that when no animal of a dyad is isolated prior to the SIT the duration of social contact is significantly lower than when one animal of the dyad is previously isolated. In the latter case, increasing the social motivation in one mouse alters the duration of social contact within the dyad. Our present results highlight the influence of the housing conditions before the tests on USVs emission. Housing conditions act as a modulator of the motivational state of the animal: mice previously isolated are more motivated to interact with a conspecific in comparison with mice which were previously housed in groups. These data would fit with previous works showing that social reward strongly modulates the emission of USVs [1], [30], [34], [37], [38] and that socially experienced male mice produce fewer syllables than inexperienced ones [22]. Animals have to be motivated for social contact (by previous social isolation in our case) and have to be rewarded by such contact to emit calls [1]. USVs emitted by adult male mice, with specific acoustic features associated with social contact, may therefore reflect social utility, not necessarily related with reproductive [11] or aggressive purposes.

A high social motivation also seems to generate a significant correlation between the number of calls and the duration of contact. In SIT-isolated mice, more than 80 per cent of the three main call types were emitted during contact. This was not the case for SIT-grouped mice, suggesting that the motivation of one animal of the dyad for engaging in a social interaction is a positive modulator of the emission of USVs. Our results extended what has been found in adolescent and female mice for which the number of calls was positively correlated to the time spent in social investigation [18], [37]. Social motivation also appeared to modulate the duration of the calls, but the direction of the effect varied according to the paradigm used. Indeed, within the SIT, mice previously isolated emitting longer calls than mice previously maintained in groups. In contrast, within the novelty exploration context, mice previously isolated emitting shorter calls than mice previously maintained in groups. The reason and the mechanisms of the influence of social motivation in the novelty exploration context remain to be investigated.

### Caveats and perspectives

The main caveat of our current study is to be unable to discriminate which of the two mice vocalized during social interaction, or whether both of them did. We ruled out using devocalization to examine the quantitative contribution of both mice to the detected calls since this intervention would certainly perturb the social interactions. We never, or very rarely, recorded overlapping calls (in the “composite” class only), which would show without any doubt that both mice vocalized at the same time. However, they could also vocalize at different times. Without an individual vocal signature or a way to estimate -either visually or by mathematical method- which of them vocalize, we have to consider them as a dyad, like other authors do [12], [39]–[42]. Only recently are studies investigating kinship and individual specificity as well as call convergence in ultrasonic vocalizations [43], [44]. More specifically on individual signatures, Hoffmann and colleagues [43] focused on courtship vocalizations of wild house mice and highlighted signatures for individuality only in a few call types, with some overlap between individuals. However, limitations of this acoustical method for discriminating caller identity might come from three points. First, individuality in laboratory mice might be much more limited in comparison with wild house mice given their relative genetic homogeneity. Second, a vocal signature may be identified in some call types but not others. Third, call individuality might be different between the different paradigms (male courtship versus same sexe social interaction). According to these promising results, future studies should concentrate on a refinement of the acoustic analyses and on the development of other methods (e.g., video analyses, signal analysis with triangulation).

We proposed here a first insight in the involvement of emotional and motivational individual states on the emission of ultrasonic vocalizations in adult male mice and a way to understand how these calls could be related to social behaviors. This framework is important to explore the mouse models of social disorders such as autism spectrum disorders, that in some cases, display impaired social and acoustic communication skills [5], [15], [45]–[47]. The temporal sequence of these two types of signals should allow to determine whether calls are emitted at the beginning (initiation of contact), or throughout the interaction (maintenance of contact). New paradigms including playback experiments should also provide information on whether mice distinguish the saliency of calls (broadcasted in unexpected situations) and whether mice discriminate the emotional content of calls.

## Supporting Information

Figure S1 **Details of peak frequency (min, max, start and end) for each call.** Representation of the four peak frequencies for each calls types in the five different conditions.
(EPS)

Table S1 **Details of statistical results for group effects and for paired comparisons of total number of calls for each call types in the five different conditions.**
(DOCX)

Table S2 **Details of statistical results for group effects and for paired comparisons of call duration for each call types in the five different conditions.**
(DOCX)

Table S3 **Details of statistical results for group effects and for paired comparisons in each peak frequency in the five different conditions.**
(DOCX)
