# Supplementary material for: Adult Male Mice Emit Context-Specific Ultrasonic Vocalizations That Are Modulated by Prior Isolation or Group Rearing Environment
Source: PLoS One. 2012 Jan 6;7(1):e29401. doi: 10.1371/journal.pone.0029401 (PMC3253078; doi:10.1371/journal.pone.0029401)
Supplement: Table S1 — Details of statistical results for group effects and for paired comparisons of total number of calls for each call types in the five different conditions. (DOCX) [file pone.0029401.s002.docx]

**Table S1.** Statistical results for group effect (Kruskall & Wallis) and for paired comparisons of total number of calls (Mann-Whitney), or paired comparisons for each call types (Chi-square (X²) test) in different types of conditions. Significance threshold was set at p<0.005.

| A | ***Total number of calls*** *(Group effect: H 4 = 34.303, P = <0. 0001)* | | | | | | | |
| --- | --- | --- | --- | --- | --- | --- | --- | --- |
|  | SIT-grouped (N=8) | | Exploration-grouped (N=8) | | Exploration-isolated (N=15) | | Restraint  (N=16) | |
| SIT-isolated (N=17) | U=29.5, P=0.024 | | U=15, P=0.002 | | U=11.5,P=<0.0001 | | U=12.5, P=<0.0001 | |
| SIT-grouped (N=8) | - | | U=24, P=0.400 | | U=21, P=0.0118 | | U=24, P=0.014 | |
| Exploration-grouped (N=8) | - | | - | | U=17.5, P=0.0061 | | U=27, P=0.023 | |
| Exploration-isolated (N=15) | - | | - | | - | | U=117, P=0.905 | |
| B | ***Short*** *(Group effect: H 4 = 13.80, P = 0. 008)* | | | | | | |  |
|  | SIT-grouped (N=8) | Exploration-grouped (N=8) | | Exploration-isolated (N=15) | | Restraint  (N=16) | |  |
| SIT-isolated (N=17) | X²=8.46, P=0.003 | X²=4.06,P=0.043 | | X²=3.42,P=0.064 | | X²=1.13,P=0.286 | |  |
| SIT-grouped (N=8) | - | X²=0.83,P=0.361 | | X²=1.17,P=0.279 | | X²=3.47,P=0.062 | |  |
| Exploration-grouped (N=8) | - | - | | X²=0.03,P=0.866 | | X²=0.91,P=0.33 | |  |
| Exploration-isolated (N=15) | - | - | | - | | X²=0.62,P=0.43 | |  |
| C | ***Composite*** *(Group effect: H 4 = 44.25, P = <0. 0001)* | | | | | | |  |
|  | SIT-grouped (N=8) | Exploration-grouped (N=8) | | Exploration-isolated (N=15) | | Restraint  (N=16) | |  |
| SIT-isolated (N=17) | X²=0.95,P=0.329 | X²=7.54,P=0.006 | | X²=27.30,P<0.0001 | | X²=38.53,P<0.0001 | |  |
| SIT-grouped (N=8) | - | X²=3.62,P=0.057 | | X²=21.13,P<0.0001 | | X²=31.87,P<0.0001 | |  |
| Exploration-grouped (N=8) | - | - | | X²=8.85,P=0.003 | | X²=17.22,P<0.0001 | |  |
| Exploration-isolated (N=15) | - | - | | - | | X²=1.61,P=0.204 | |  |
| D | ***Downward*** *(Group effect: H 4 = 14.29, P = 0. 0064)* | | | | | | |  |
|  | SIT-grouped (N=8) | Exploration-grouped (N=8) | | Exploration-isolated (N=15) | | Restraint  (N=16) | |  |
| SIT-isolated (N=17) | X²=2.20,P=0.138 | X²=0.37,P=0.544 | | X²=2.40,P=0.121 | | X²=1.22,P=0.27 | |  |
| SIT-grouped (N=8) | - | X²=0.89,P=0.346 | | X²=0.007,P=0.93 | | X²=0.20,P=0.655 | |  |
| Exploration-grouped (N=8) | - | - | | X²=1.04,P=0.308 | | X²=0.272,P=0.602 | |  |
| Exploration-isolated (N=15) | - | - | | - | | X²=0.28,P=0.595 | |  |

| E | ***Upward*** *(Group effect: H 4 = 38.008, P = <0. 0001)* | | | |
| --- | --- | --- | --- | --- |
|  | SIT-grouped (N=8) | Exploration-grouped (N=8) | Exploration-isolated (N=15) | Restraint  (N=16) |
| SIT-isolated (N=17) | X²=6.15,P=0.013 | X²=14.87,P=0.0001 | X²=17.62,P<0.0001 | X²=14.14,P=0.0001 |
| SIT-grouped (N=8) | - | X²=3.286,P=0.07 | X²=5.66,P=0.017 | X²=2.77,P=0.096 |
| Exploration-grouped (N=8) | - | - | X²=0.98,P=0.32 | X²=0.03,P=0.847 |
| Exploration-isolated (N=15) | - | - | - | X²=1.27,P=0.258 |
| F | ***One frequency jump*** *(Group effect: H 4 = 29.67, P = <0. 0001)* | | | |
|  | SIT-grouped (N=8) | Exploration-grouped (N=8) | Exploration-isolated (N=15) | Restraint  (N=16) |
| SIT-isolated (N=17) | X²=0.80,P=0.369 | X²=0.31,P=0.578 | X²=7.35,P=0.007 | X²=14.99,P=0.0001 |
| SIT-grouped (N=8) | - | X²=0.12,P=0.731 | X²=3.56,P=0.06 | X²=10.07,P=0.0015 |
| Exploration-grouped (N=8) | - | - | X²=4.86,P=0.027 | X²=11.85,P=0.0006 |
| Exploration-isolated (N=15) | - | - | - | X²=2.50,P=0.113 |
| G | ***Modulated*** *(Group effect: H 4 = 22.42, P = 0. 0002)* | | | |
|  | SIT-grouped (N=8) | Exploration-grouped (N=8) | Exploration-isolated (N=15) | Restraint  (N=16) |
| SIT-isolated (N=17) | X²=2.22,P=0.135 | X²=0.51,P=0.474 | X²=6.88,P=0.008 | X²=8.11,P=0.004 |
| SIT-grouped (N=8) | - | X²=0.64,P=0.422 | X²=1.83,P=0.176 | X²=2.77,P=0.095 |
| Exploration-grouped (N=8) | - | - | X²=4.16,P=0.041 | X²=5.30,P=0.021 |
| Exploration-isolated (N=15) | - | - | - | X²=0.18,P=0.671 |
| H | ***Frequency jumps*** *(Group effect: H 4 = 23.99, P = <0. 0001)* | | | |
|  | SIT-grouped (N=8) | Exploration-grouped (N=8) | Exploration-isolated (N=15) | Restraint  (N=16) |
| SIT-isolated (N=17) | X²=4.14,P=0.041 | X²=2.83,P=0.09 | X²=4.12,P=0.042 | X²=6.34,P=0.011 |
| SIT-grouped (N=8) | - | X²=0.212,P=0.645 | X²=0.0001,P=0.99 | X²=0.87,P=0.35 |
| Exploration-grouped (N=8) | - | - | X²=0.20,P=0.651 | X²=1.59,P=0.206 |
| Exploration-isolated (N=15) | - | - | - | X²=0.88,P=0.35 |
| I | ***U-Shape*** *(Group effect: H 4 = 21.25, P = 0. 0003)* | | | |
|  | SIT-grouped (N=8) | Exploration-grouped (N=8) | Exploration-isolated (N=15) | Restraint  (N=16) |
| SIT-isolated (N=17) | X²=0.99,P=0.32 | X²=1.36,P=0.243 | X²=1.36,P=0.243 | X²=1.36,P=0.243 |
| SIT-grouped (N=8) | - | X²=0.14,P=0.71 | X²=0.14,P=0.71 | X²=0.14,P=0.71 |
| Exploration-grouped (N=8) | - | - | - | - |
| Exploration-isolated (N=15) | - | - | - | - |
|  |  |  |  |  |
| J | ***Chevron*** *(Group effect: H 4 = 17.48, P = 0. 0016)* | | | |
|  | SIT-grouped (N=8) | Exploration-grouped (N=8) | Exploration-isolated (N=15) | Restraint  (N=16) |
| SIT-isolated (N=17) | X²=0.50,P=0.48 | X²=0.68,P=0.407 | X²=0.68,P=0.407 | X²=0.68,P=0.407 |
| SIT-grouped (N=8) | - | X²=0.072,P=0.788 | X²=0.072,P=0.788 | X²=0.072,P=0.788 |
| Exploration-grouped (N=8) | - | - | - | - |
| Exploration-isolated (N=15) | - | - | - | - |
