# Supplementary material for: Adult Male Mice Emit Context-Specific Ultrasonic Vocalizations That Are Modulated by Prior Isolation or Group Rearing Environment
Source: PLoS One. 2012 Jan 6;7(1):e29401. doi: 10.1371/journal.pone.0029401 (PMC3253078; doi:10.1371/journal.pone.0029401)
Supplement: Table S2 — Details of statistical results for group effects and for paired comparisons of call duration for each call types in the five different conditions. (DOCX) [file pone.0029401.s003.docx]

**Table S2.** Statistical results for group effect (Kruskall & Wallis) and for paired comparisons (Mann-Whitney) in each duration of call types. Significance threshold was set at p<0.005.

| A | **Call duration** (Group effect: H 4 = 36.51, P = <0. 0001) | | | | | | |  |
| --- | --- | --- | --- | --- | --- | --- | --- | --- |
|  | SIT-grouped (N=8) | | Exploration-grouped (N=8) | | Exploration-isolated (N=15) | Restraint  (N=16) | |  |
| SIT-isolated (N=17) | U=112,P<0.0001 | | U=120, P=0.00147 | | U=245,  P<0.0001 | U=234, P=0.0002 | |  |
| SIT-grouped (N=8) | - | | U=36, P=0.7209 | | U=112, P=0.0002 | U=68, P=0.834 | |  |
| Exploration-grouped (N=8) | - | | - | | U=10,P=0.0005 | U=56, P=0.652 | |  |
| Exploration-isolated (N=15) | - | | - | | - | U=18, P<0.0001 | |  |
| B | ***Short*** *(Group effect: H 4 = 36.27, P<0.0001)* | | | | | | | |
|  | SIT-grouped (N=8) | Exploration-grouped (N=8) | | Exploration-isolated (N=15) | | | Restraint  (N=16) | |
| SIT-isolated (N=17) | U=104, P=0.03 | U=108, P=0.018 | | U=248,P<0.0001 | | | U=188,P=0.0052 | |
| SIT-grouped (N=8) | - | U=51,P=0.049 | | U=117,P<0.0001 | | | U=55,P=0.973 | |
| Exploration-grouped (N=8) | - | - | | U=16,P=0.0032 | | | U=21,P=0.015 | |
| Exploration-isolated (N=15) | - | - | | - | | | U=6,P<0.0001 | |
| C | ***Composite*** *(Group effect: H 4 = 43.63, P<0.0001)* | | | | | | | |
|  | SIT-grouped (N=8) | Exploration-grouped (N=8) | | Exploration-isolated (N=15) | | | Restraint  (N=16) | |
| SIT-isolated (N=17) | U=93.5,P=0.0042 | U=118,P<0.0001 | | U=221,P<0.0001 | | | U=238,P<0.0001 | |
| SIT-grouped (N=8) | - | U=44,P=0.0721 | | U=97,P=0.0007 | | | U=93,P=0.011 | |
| Exploration-grouped (N=8) | - | - | | U=10,P=0.00055 | | | U=76,P=0.489 | |
| Exploration-isolated (N=15) | - | - | | - | | | U=26,P<0.0001 | |
| D | ***Upward*** | | | | | | | |
|  | SIT-grouped (N=8) | Exploration-grouped (N=8) | | Exploration-isolated (N=15) | | | Restraint  (N=16) | |
| SIT-isolated (N=17) | U=30,P=0.3587 | - | | - | | | - | |
| SIT-grouped (N=8) | - | - | | - | | | - | |
| Exploration-grouped (N=8) | - | - | | - | | | - | |
| Exploration-isolated (N=15) | - | - | | - | | | - | |

| E | ***Modulated*** | | | |
| --- | --- | --- | --- | --- |
|  | SIT-grouped (N=8) | Exploration-grouped (N=8) | Exploration-isolated (N=15) | Restraint  (N=16) |
| SIT-isolated (N=17) | U=55,P=0.1418 | - | - | - |
| SIT-grouped (N=8) | - | - | - | - |
| Exploration-grouped (N=8) | - | - | - | - |
| Exploration-isolated (N=15) | - | - | - | - |
| F | ***One frequency jump*** | | | |
|  | SIT-grouped (N=8) | Exploration-grouped (N=8) | Exploration-isolated (N=15) | Restraint  (N=16) |
| SIT-isolated (N=17) | U=78,P=0.0266 | U=101,P=0.0014 | - | - |
| SIT-grouped (N=8) | - | U=31,P=0.1807 | - | - |
| Exploration-grouped (N=8) | - | - | - | - |
| Exploration-isolated (N=15) | - | - | - | - |
