# Supplementary material for: Adult Male Mice Emit Context-Specific Ultrasonic Vocalizations That Are Modulated by Prior Isolation or Group Rearing Environment
Source: PLoS One. 2012 Jan 6;7(1):e29401. doi: 10.1371/journal.pone.0029401 (PMC3253078; doi:10.1371/journal.pone.0029401)
Supplement: Table S3 — Details of statistical results for group effects and for paired comparisons in each peak frequency in the five different conditions. (DOCX) [file pone.0029401.s004.docx]

**Table S3.** Statistical results for group effect (Kruskall & Wallis) and for paired comparisons (Mann-Whitney) in each peak frequency. Significance threshold was set at p<0.005.

| A | ***Peak frequency min*** *(Group effect: H 4 = 42.82, P = <0. 0001 )* | | | |
| --- | --- | --- | --- | --- |
|  | SIT-grouped (N=8) | Exploration-grouped (N=8) | Exploration-isolated (N=15) | Restraint  (N=16) |
| SIT-isolated (N=17) | U=50, P=0.294 | U=2, P=0.0001 | U=3, P<0.0001 | U=2, P<0.0001 |
| SIT-grouped (N=8) | - | U=16, P=0.0929 | U=22, P=0.0142 | U=9, P=0.0008 |
| Exploration-grouped (N=8) | - | - | U=42, P=0.2453 | U=16, P=0.0033 |
| Exploration-isolated (N=15) | - | - | - | U=43, P=0.0023 |
| B | ***Peak frequency max*** *(Group effect: H 4 = 38.47, P = <0. 0001 )* | | | |
|  | SIT-grouped (N=8) | Exploration-grouped (N=8) | Exploration-isolated (N=15) | Restraint  (N=16) |
| SIT-isolated (N=17) | U=23.5, P=0.0095 | U=7, P=0.0004 | U=4, P<0.0001 | U=3, P<0.0001 |
| SIT-grouped (N=8) | - | U=21, P=0.248 | U=23, P=0.0169 | U=17, P=0.004 |
| Exploration-grouped (N=8) | - | - | U=33.5, P=0.0872 | U=29, P=0.0321 |
| Exploration-isolated (N=15) | - | - | - | U=102, P=4768 |
| C | ***Peak frequency start*** *(Group effect: H 4 = 42.34, P = <0. 0001)* | | | |
|  | SIT-grouped (N=8) | Exploration-grouped (N=8) | Exploration-isolated (N=15) | Restraint  (N=16) |
| SIT-isolated (N=17) | U=37.5, P=0.075 | U=6, P=0.0003 | U=3, P<0.0001 | U=2, P<0.0001 |
| SIT-grouped (N=8) | - | U=19, P=0.172 | U=25, P=0.023 | U=9, P=0.0008 |
| Exploration-grouped (N=8) | - | - | U=39, P=0.175 | U=13, P=0.001 |
| Exploration-isolated (N=15) | - | - | - | U=52, P=0.007 |
| D | ***Peak frequency end*** *(Group effect: H 4 = 44.50, P = <0. 0001)* | | | |
|  | SIT-grouped (N=8) | Exploration-grouped (N=8) | Exploration-isolated (N=15) | Restraint  (N=16) |
| SIT-isolated (N=17) | U=32, P=0.036 | U=3, P=0.0002 | U=25, P<0.0001 | U=1, P<0.0001 |
| SIT-grouped (N=8) | - | U=18, P=0.141 | U=25, P=0.023 | U=6, P=0.0004 |
| Exploration-grouped (N=8) | - | - | U=39, P=0.175 | U=16, P=0.003 |
| Exploration-isolated (N=15) | - | - | - | U=39, P=0.001 |
